# Lab-on-chip technologies for space research — current trends and prospects

**DOI:** 10.1007/s00604-023-06084-4

**Published:** 2023-12-14

**Authors:** Agnieszka Krakos

**Affiliations:** Department of Microsystems, Wroclaw University of Science and Technology, Janiszewskiego 11/17, 50-372 Wroclaw, Poland

**Keywords:** Microfluidics, Nanofluidics, Lab-on-chip, Space biology, Microfluidic payload, Bio-nanosatellite

## Abstract

The in-depth analysis concerning application of microfluidic instruments for space biology research is presented. The article focuses on recently investigated key scientific fields, i.e., lab-on-chips applied to the biomedical studies performed in the (1) International Space Station and (2) CubeSat nanosatellites. The paper presents also the lab-on-chip devices that were fabricated with a view to future space biology research and to those that to date have been solely been tested under Earth laboratory conditions and/or simulated microgravity environments. NASA and ESA conceptual mission plans for future are also mentioned, concerning for instance “tissue chips” and the ESA-SPHEROIDS campaign. The paper ends with final conclusions and future perspectives regarding lab-on-chip application in the space biology sector and its impact on novel biomedical and pharmaceutical strategies.

**Graphical Abstract:**

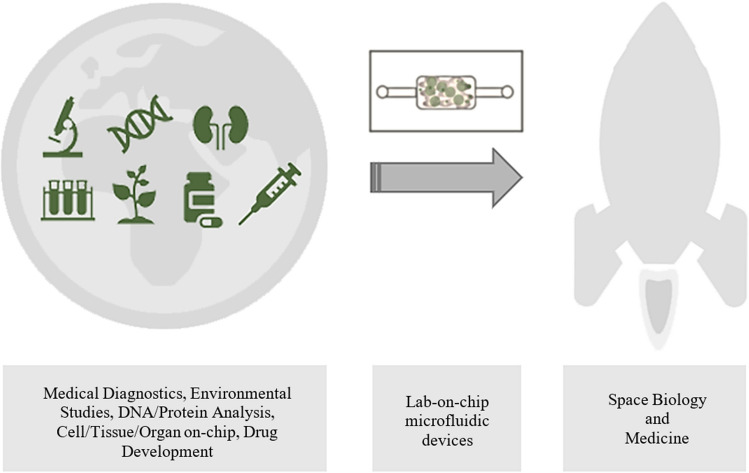

## Introduction

**Fig. 1:**
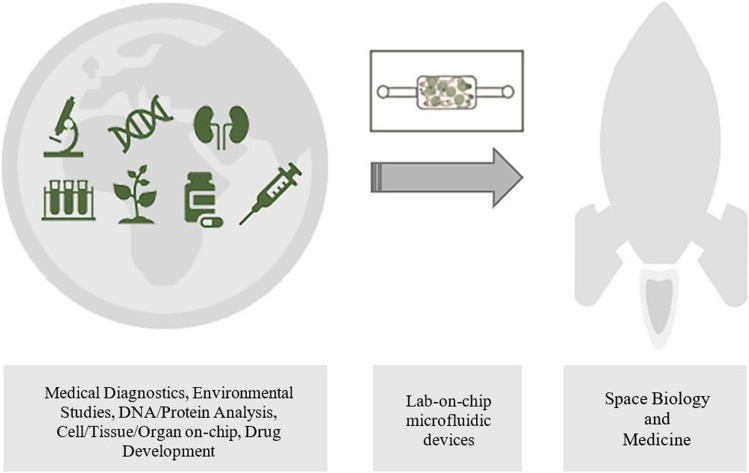
Lab-on-chip: research areas

Lab-on-chip (LOC) technology has bloomed in the early 1990s to become a significant part of the *life science* branch. Initially, the major development directions and, thus applications of LOCs have related to chemistry, nucleic/protein analysis and generally biomedicine [1–5]. However, recent advances in the field ignites towards novel areas, i.e., space science astrobiological research (Fig. 1).

The key features of microfluidic-related LOCs cover low reagent consumption, reduced contamination risks, high throughput screening, short analysis and response time, and automation possibilities. These and other advantages make lab-on-chip instruments to play an important role in the bio-engineering research [6–8]. Although it is still an adolescent discipline, requiring much attention and interdisciplinary scientific cooperation, many microfluidic devices have become an inherent part of our daily lives, indicating towards further development for healthcare and personalized medicine, also in the context of, so called, space medicine, and basic tools for astronauts diagnostics [9–13].

**Fig. 2:**
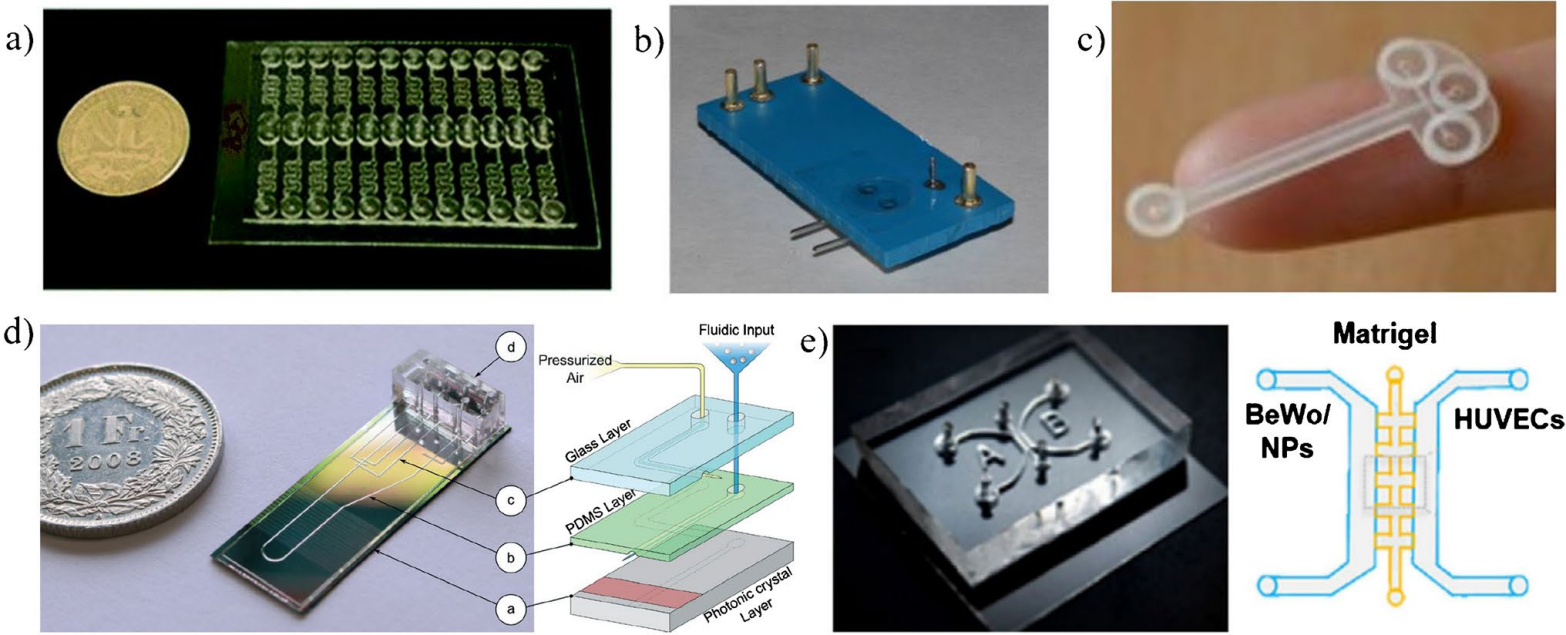
The examples of LOCs fabricated out of **a** polycarbonate and PDMS [26], **b** ceramics [10], **c** 3D printable photocurable resins [18], **d** silicon, glass, and PDMS [25], **e** PDMS and Matrigel hydrogel [4]

Fabrication of LOCs has its origin in microelectronics and silicon micromachining techniques, dating back to the early 1950s [14–16]. Micro-nanoscale structures of high precision can be obtained utilizing techniques of photolithography and soft-lithography, chemical etching, micromilling, bonding, or lastly gaining in popularity 3D printing and 3D bio-printing [17–20]. Different materials are employed [21–24], which typically are silicon, glass (borosilicate, Foturan), ceramics (low temperature cofired ceramics – LTCC) and polymers. The last of the aforementioned group is the broadest one, since it encompasses both materials which are (1) well-known in microfluidics — polystyrene (PS), polydimethylosiloxane (PDMS), cyclic olefin copolymer (COC), (2) popular with 3D printing technique — acrylonitrile butadiene styrene (ABS), polylactide (PLA), and (3) 3D bio-printing, like hydrogel inks of diverse, often unique composition, basing on alginate or agar components (Fig. 2).

With a view to the diverse material and techniques availability, it should be emphasized that LOCs in many cases constitute hybrid, sandwiched-type structures, and different materials and technological processed are employed for the fabrication of a single chip [25–27]. In the case of PDMS LOCs (the most common ones), often solution is to use a glass cover, connected impermanently with the polymer substrate which notably enhances optical detection and ensures appropriate assembly of the microfluidic connectors. As for the ground-based studies “all holds are allowed” to get the most out of the structure and provide the best performance, the situation may become a little complicated when the research is going to be done in space, especially with a view to limited experiment control.

Microfluidics with its fundamental feature of laminar microflow, and slow but controlled reagents drift based solely on the diffusion effects, is from the definition adequate for microgravity applications. Other advantages of LOC instrumentation, i.e., miniaturization aspect, low-weight and low-power, significantly reduces the costs and open the way towards many space applications, also for the deep space missions. Nevertheless, the development of fully integrated microfluidic systems, able to work completely independently, in a fully automated and remotely controlled way is still a big challenge. Herein, necessity to construct a laboratory payload in a form of a totally self-contained system, failure-free operation and consecutive signal transfer is not the only problem. A “bottleneck” in this regard may also constitute a technology flow, employed for the LOC and entire platform fabrication. Materials and encapsulation methods must meet the specific demands required for space systems. One of the most important issue, especially noteworthy with microfluidic structures, is to fulfil the low outgassing criteria and inhibit excessive liquid evaporation [28]. Small air bubbles which are immersed in fluid do not constitute a serious problem in the Earth conditions. Real danger appears in space, where the pressure drop can enlarge the bubbles and cause a notable microflow blockade [29]. On that basis, any methods which could minimize or eliminate this problem are highly desired. As the recent microfluidics trend is mostly related to polymer (PDMS) structures which cannot be appropriately operated in space, novel solutions need to be proposed. Possibly, return to the standard microengineering materials, i.e., silicon/glass for the LOC substrates which can be permanently joint with bonding methods, and thus, perfectly encapsulated, are one of the prospective strategy for in-space research [30]. As reference point herein can be treated silicon-glass MEMS (micro-electro-mechanical system) micropropulsion devices (micronozzles, cold-gas microthrusters) successfully used for the space missions since 1960s and distributed recently by many space companies as regular solutions [31].

The role of microfluidic instruments for space applications each year is being more and more visible. Except for the diagnostic tools used for monitoring of astronauts’ health and living conditions [32–34], one of the most powerful branch which can be distinguished now is the use of lab-on-chip platforms for astrobiological research in microgravity and radiation conditions. Fundamentals of this investigation lay in atypical behaviour of microbial objects in space, being a result of 0-gravity force. Literature reports describe, e.g., enhanced protein crystal growth [35, 36] or 3D spheroid structure formation of cancer cell lines in weightlessness state [37, 38] which have been studied both in International Space Station (ISS) or on a satellites (CubeSats) boards. On the other hand, increasingly common is to use microgravity simulators (Clinostats, Random Positions Machines, Rotary Wall Vessels) prior to space experimentation to imitate weightlessness state to some extend and provide important baselines prior to the mission [39].

In this review, the major focus is put on the description of the lab-on-chip platforms that have been used or are going to be used as laboratory payloads in space (ISS, satellites) and deep space missions, in the context of microgravity and radiation influence onto widely understandable life. The analysis covers also the concepts of microfluidic assisted missions and LOC instruments that have been investigated solely in simulated microgravity conditions and have not been verified in real space-based environments. As a final conclusion, perspective for future is outlined, basically about the impact regarding further development of space medicine, biology and pharmaceutics utilizing lab-on-chip technology.

## LOC platforms towards potential, future space biology research

As the microfluidics has a potential to accelerate the space science notably, the literature on the subject reports on both single lab-on-chip structures and whole LOC platforms fabricated with a view to future space applications.

**Fig. 3:**
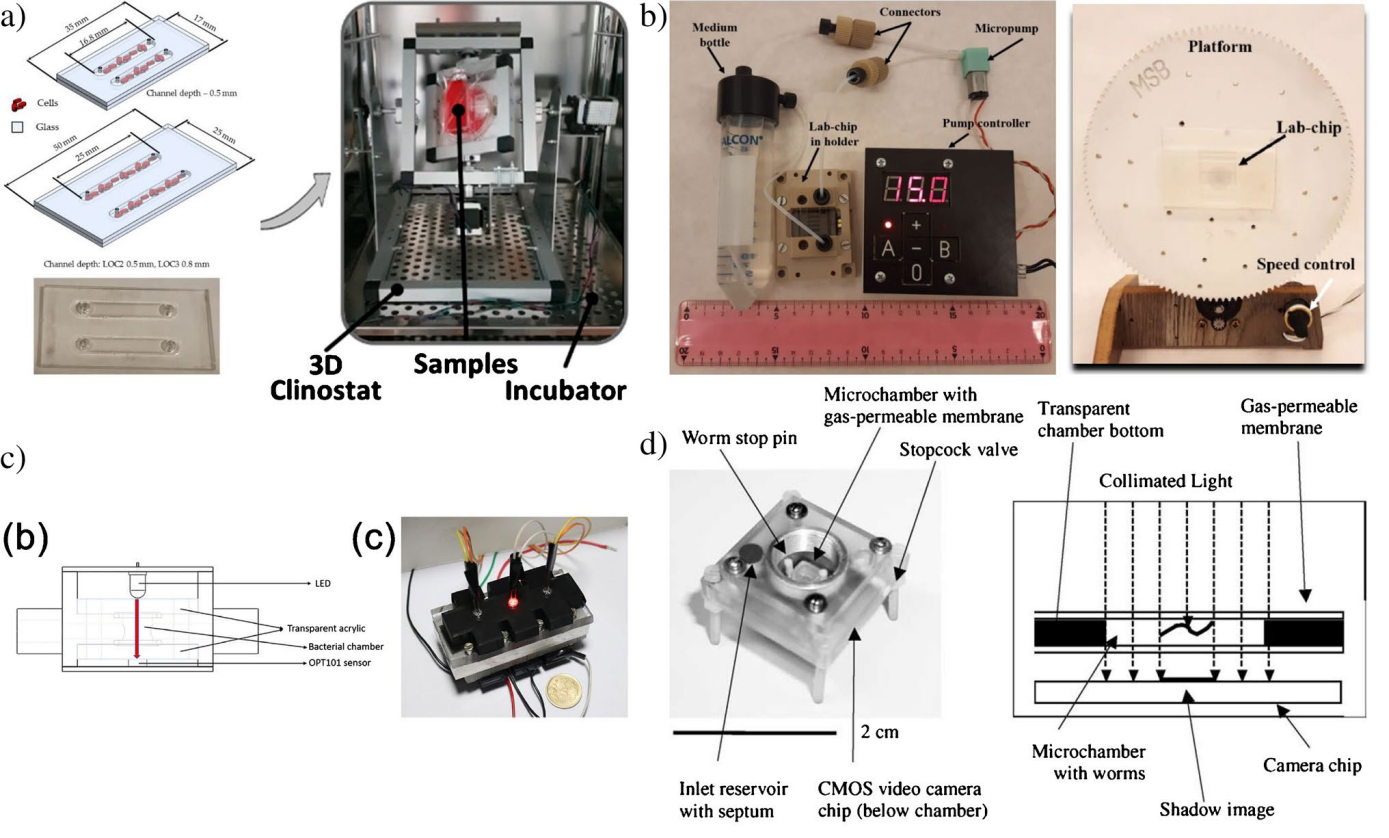
Microfluidic structures developed with a view to potential future space applications: **a** all-glass LOC for culturing of human keratinocytes HaCaT and melanoma cells A375 utilizing RPM [40], **b** photograph and schematic diagram of the worm imager. Nematodes in a microchamber are illuminated with an LED and cast a shadow onto a CMOS video camera chip attached at the bottom of the chamber [44], **c** photograph of the culturing device with a scheme representing operational principle for OD measurement: LED light source is used to illuminate the sample within the bacterial chamber, transmitted light intensity is measured by a photodiode (OPT101 sensor) [49], **d** LOC platform for microscopic fungi culture (*F. culmorum*) tested in simulated microgravity conditions with RWV [50]

Beginning with the LOCs simply, in the article [40], the application of the all-glass chip is shown as the new tool ensuring culturing of human keratinocytes HaCaT and melanoma cells A375 under simulated microgravity conditions utilizing 3D-clinostat (Fig. 3a). The chip was fabricated utilizing standard microengineering techniques, i.e., xurography, deep wet chemical etching and high temperature fusion bonding of glass substrates. Evaluation of cells viability, proliferation, and morphology, as well as mitochondrial and caspase activity, revealed that HaCaT and A375 cells are highly susceptible to simulated microgravity and further research in real space environments is needed.

In the paper [41], the authors present a micro-scale fluorescent-activated cell sorter (μFACS) ensuring live-dead cells counts and sorting based on the fluorescence characteristics. Microcontroller operation of high-speed microvalves provides grouping of the cells in specific chambers —waste or collection. Recently, the system is being adapted to fit the nanosatellite unit and conduct further biological experiments in Low Earth Orbit (LEO).

W. Wang et al. propose the microfluidic culturing platform utilizing genetic engineered bacteria as a bioindicator of space radiation doze [42]. This solution seems as substantial improvement over commonly used in ISS physical dosimeters (e.g., thermo-luminescence detector — TLDs, DOSimetry TELescope — DOSTEL), and can provide rapid evaluation of physiological radiation influence, which is necessary in the context of astronauts’ health during long-term space flight missions.

A novel solution of microfluidic chip for space applications was recently developed by Y. Chen et al., ensuring co-culture and study of intercellular cells interactions during potential space flights [43]. The background of these studies lies in astronauts’ exposure to space environments and finding the model that would fit the biological interplay between the human cells of different origin at these conditions. The authors improved the lab-on-chip technology and provided significant surface modification based on plasma and ultraviolet treatment for the selected microfluidic substrates, i.e., PS and PET. Physiological cells growth was provided for both adherent and suspension objects on-chip showing appropriate structure performance in terms of biocompatibility and stability.

D. Lange et al. report on the development of shadow imaging device ensuring behavioural analysis of the model microorganisms — nematode *Caenorhabditis elegans* [44]. The selection of this biological object was not an accidental choice herein — nematodes are well known creatures used for experimental genetics, radiobiology, and aging studies which can be of great importance in the case of future space applications [45–47]. A microscale lab-on-chip device was developed to fit the potential miniaturization payload constraints. A “worm imager” utilizing LED illumination and a shadow that casts onto CMOS camera sensor was used as a compact vision system ensuring nematodes monitoring on-chip (Fig. 3b).

On other hand, some crystallography research was done recently with regard to space outlook. M. Maeki et al. report on the microfluidic device fabrication utilizing laser confocal microscopy for the real-time crystal growth measurements [48]. As shown in the paper, 20-µm and 30 µm-deep PDMS microwells were used to develop ultra-fast and high-quality protein crystals. Results obtained for the 20-µm cavity were recognized as similar to the ISS experimentation, which suggest almost perfect microgravity imitation, thanks to microfluidics. Moreover, potential use of this structure in space conditions may further enhance the crystallization effect and put a new light onto space pharma research.

Other paper [49] shows a fully featured lab-on-chip platform, proposed to study bacteria *Sporosarcina pasteurii* growth in space environments (Fig. 3c). The platform was equipped with all the subsystems providing autonomous and real-time measurements of bacteria activity based on optical density (OD) monitoring, as well as post log-phase optical and scanning electron microscopy (SEM). The performance of the device was successfully verified in the conditions imitating spaceflight, i.e., multiple temperatures, pressures, and under different device orientations.

Interesting research was also done towards long-term fungi cultivation —*Fusarium culmorum* on-chip in space environments [50]. Special construction of all-glass LOC was proposed with pulse-based media flow system to ensure “flooding” of the cell culturing chamber directly in space. This small scale device can be used both in ISS, as well as for the nanosatellite payloads. The experiments run under this research showed appropriate performance of all the LOC platform components and physiological development of *F. culmorum*. Moreover, experiments in simulated microgravity conditions utilizing Rotary Wall Vessel were conducted and enhanced mycelium growth of fungi was observed, which can propel further investigation of this species in space (Fig. 3d).

In the paper [51], Krakos et al. described the potential implementation of biological mission with cancer cells utilizing microfluidic platform. The main goal of the studies was to fabricate the most universal LOC, which would allow for cancer cells development of different origin in ambient temperature. The research was dictated by the problem which is faced before the launch of the rockets to the ISS. Namely, nanosatellites structures are held in hangar, in ambient temperature and no life-support systems can be activated. The authors confirmed that long-term culturing of cancer cells in such harsh conditions can be achieved with microfluidics that may extend the space research of these sensitive bio-objects on CubeSat boards shortly.

An interesting idea of lab-on-chip system for potential space applications was also presented by D. Touchette et al., showing microfluidic microbial activity microassay (μMAMA) device [52]. The goal of the studies was to evaluate detection capabilities of the LOC towards extant microbial life. Based on the redox dyes, e.g., AlamarBlue with buffer staining, the metabolic activity of the astrobiological-related samples was identified from the fewest microbial cells of 102 cells/mL. Performance of the device was also verified under different pH, temperature, salinity, and perchlorate conditions, and with a view a Mars regolith simulant (MMS-2), showing appropriate operation.

Coming into the scope of the fully-featured and self-contained microfluidic platforms for the astrobiological studies, a few concepts of the payloads fitting CubeSat standards can be found in the literature recently. In brief, two research directions are distinguished as the following: (1) evaluation of the space environment influence onto terrestrial bio-samples (microgravity, radiation), and 92) deep space samples collection and identification. Regardless of the application, all of the approaches mentioned herein are presented as small scale instruments, covering miniaturization idea and the use of lab-on-chip and MEMS components.

According to the paper [53], NASA has recently established the “Starlight” program, which basic assumption is to develop a small, interstellar unit to be send outside our solar system. NASA conceptual roadmap aims at both robotic space exploration and in-depth characterization of well-known terrestrial microorganisms, with a view to their high radiation-tolerance, cryptobiotic capability and low metabolic rate, e.g., tardigrades or *C. elegans*. Low mass and energy demands of LOC instrumentation perfectly fits the deep space mission objectives. Nevertheless, authors clearly mark the needs for space-appropriate microfluidic materials, i.e., glass or thermoplastic polymers.

Staying within the path of the biological missions with small scale satellites, a few concepts of the studies in space have been recently proposed, e.g., MINERVA, GreenCube, SpectroCube, and AstroBio CubeSat.

**Fig. 4:**
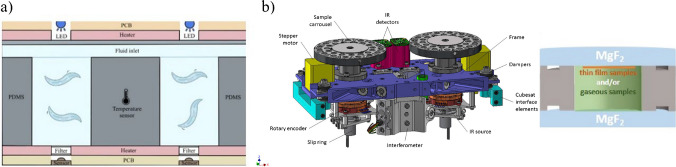
Concepts of biological missions with small satellites:) AIBO biosensor system of Minerva [54], **b** spectrometer configuration of SpectroCube with detailed view of single sample cell being a part of a sample carrousel [56]

The idea of the MINERVA mission is to study the potential radiation-tolerance of genetically manipulated *C. elegans* based on a protein-coding gene transfer [54]. The aforementioned experimentation will be conducted utilizing miniaturized biosensor system called AIBO (Autonomous Intelligent Biological Operating System) equipped with microfluidic and optical components (Fig. 4a). A 4-month cultivation of *C. elegans* exposed to deep space radiation is assumed herein. The growth rate and metabolic activity will be evaluated for both wild and modified type of *C. elegans* cultured on the microfluidic platform. Similarly, as in the case of Shenzhou-8 (SZ-8) mission, microorganisms will be launched in its Dauer state, since *C. elegans* can hibernate through controlled starvation. When the nanosatellite will reach the cis-lunar orbit, *C. elegans* will be stimulated to growth by flooding with the nutrient, similarly as shown in [50]. The launch of the satellite integrated with the microfluidic payload is planned for 2025.

GreenCube concept has been proposed by the Sapienza University of Rome, in cooperation with ASI (Italian Space Agency), ENEA (Italian National Agency for New Technologies, Energy and Sustainable Economic Development), and the University of Naples “Federico II” [55]. The idea of this biological satellite is to culture brassicacee species — *Lepidium sativum* (garden cress) within a miniaturized and remotely controlled biological payload. As the matter of in-orbit plants cultivation (vegetables, fruits, etc.) is of great importance in the context of manned space missions and ISS infrastructure, this project will focus on the microgreens growth in autonomous, hydroponic system.

Within the payload prepared in a form of pressurized vessel operated at 50 kPa, all the necessary components will be arranged to ensure the missions objectives. A miniature matrix of 7 × 7 units to cultivate 37 seeds will be used, as well as air, carbon dioxide and nutrient tanks. LED diode will be provided to allow for microgreens photosynthetic cycle. Cameras (VIS-band and thermal) will be applied to acquire and save the data images of the plant growth. The sensors of pressure, temperature, humidity will be used as well. In the case of the satellite bus, commercial, space-proven components will be applied. A full mission time will cover 30 days. Growth experiments will be carried out for 20 days.

SpectroCube is another European concept for microfluidic application in astrobiology and astrochemistry research with satellites [56]. The studies are going to be provided in situ at highly elliptical orbit around the Earth, thus in stronger solar UV and radiation fields. Analysis of the samples representing life detection biomarkers, such as organic molecules, will be done utilizing miniature infrared spectrometer. Special attention will be paid to monitoring the molecules degradation profiles and photostability.

SpectroCube payload will cover at least third of the CubeSat volume (the remaining space of the payload is dedicated to attitude control, on-board computer, communication, and propulsion modules). Sample containers are designed as cylindrical cells with special windows fabricated of the materials that exhibit good UV transmission properties. The windows will probably be made of magnesium fluoride (MgF_2_), calcium fluoride (CaF_2_), or potassium bromide (KBr), Fig. 4b.

As mentioned earlier, the most important element of the platform is infrared spectrometer accompanied by UV and radiation sensors (Fig. 4b). The tests on both mechanical durability and spectroscopy performance were recently done to assure the weak points prior to real space mission. Based on the results of random vibration tests, it could be concluded that the interferometer can be effectively protected from the damage and misalignment. Moreover, the spectra of the selected organic molecules (e.g., NH_3_, COH, CH_3_) and gases (CO_2_, CH_4_) that can potentially be present during the mission, were successfully identified by the spectrometer.

The project on AstroBio CubeSat was selected by ESA to be launched to MEO, into the internal Van Allen belt [57]. The satellite will be equipped with microfluidic tools to ensure investigation of immunoassay techniques exploiting chemiluminescence detection in harsh space environments. The basic aim of the mission is to assess the performance of lab-on-chip system and mitigate the potential effect of very high charged particles flux. In detail, fluid dynamics, reagents stability, readout noise, etc. will be studied in MEO and special protection solutions will be developed. For instance, appropriate temperature and pressure within the payload will be provided by hermetically sealed aluminum box shielding from radiation. In the box, also main subsystems, i.e., batteries, on-board data handling, telemetry, and active thermal control system, will be hosted. The aforementioned mission may be a significant breakthrough in the context of lab-on-chip technology and its utility in deep space missions.

It has to be also emphasized that the concepts of small scale satellites utilizing LOC instrumentation for biomedical research are becoming increasingly popular with education section as well. Herein, many interesting student ideas are presented annually at the most prestigious space science conferences — International Astronautic Congress and The Small Satellite Conference [58–60].

Summing up the section on microfluidic based instruments for the potential space science research, the interest in developing new space-oriented microscale methodologies is getting more and more visible. Touching the problem of astrobiology, astrochemistry and astropharmaceutics, the aforementioned examples present diverse lab-on-chip approaches — for cells analysis, biomarkers identification, crystal growth dynamics, or deep-space radiation cell-effects. The necessity to further develop miniaturization techniques and methodologies is certain, since the application multiplicity is rather bold and limitless. Failure-free operation of miniaturized systems and tests performed directly in space relevant environments requires however substantial financial outlay and equal global opportunities. By now, space microfluidics TRL level is still estimated as medium which notably confine its global space utility [61].

## LOC platforms used for space biology research on ISS

**Fig. 5:**
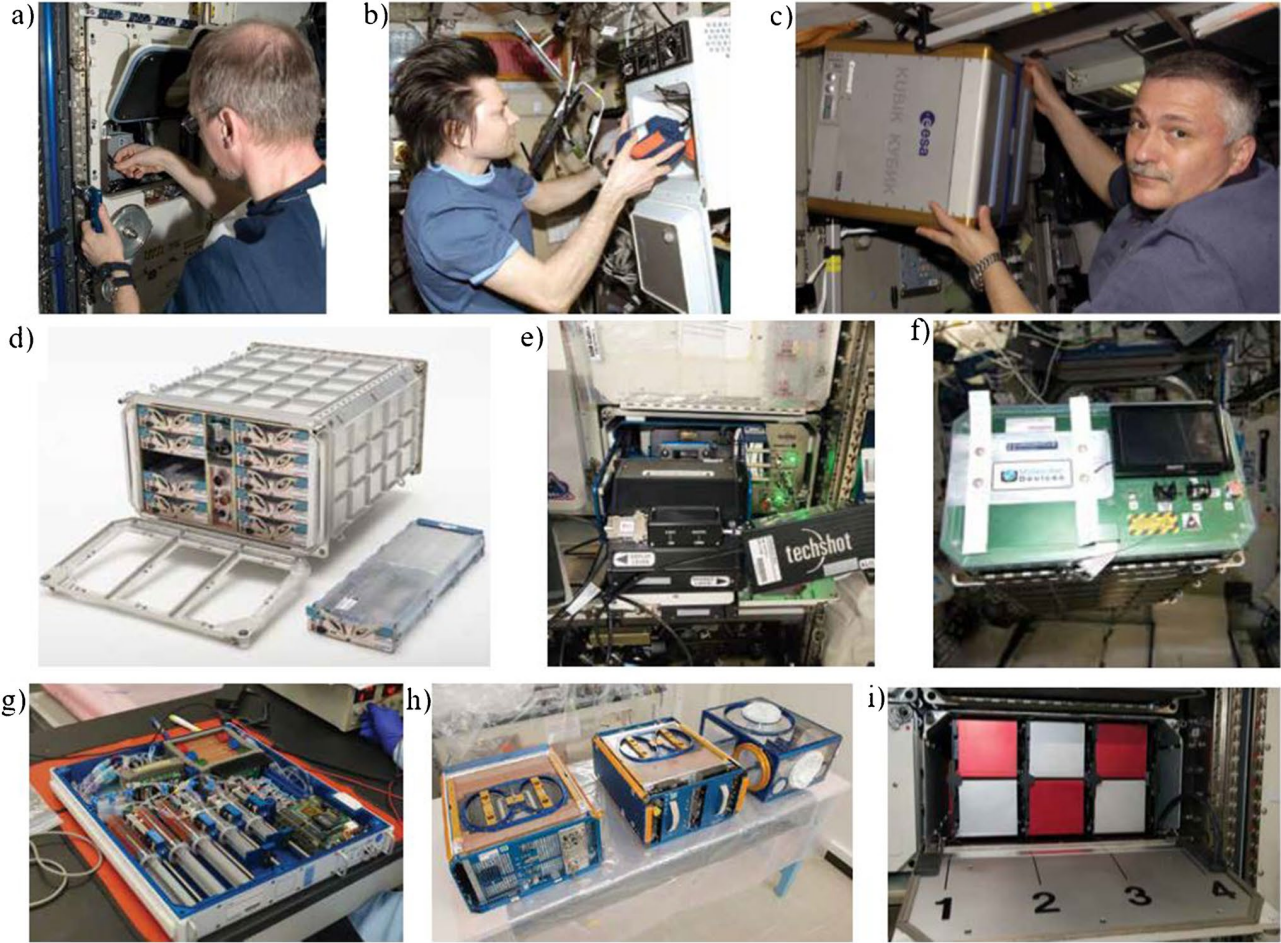
The examples of ISS facilities for biomedical research in space: **a** Cosmonaut Fyodor Yurchikhin working with a KUBIK incubator, **b** ESA astronaut Frank De Winne installing experiments in the BioLab incubator, **c** ISS crewmember Oleg Kononenko placing experiment the Kriogem-3 M Refrigerator, **d** Bioculture System Facility, **e** Bone Densitometer, **f** NanoRacks Plate Reader, **g** Osteo-4, **h** Rodent Research Hardware System, **i** TangoLab-1. Images courtesy of NASA [62]

The infrastructure of ISS is extensive and lot of facilities are available to ensure animal, plant, cellular and microbial research (Table 1., Fig. 5) [62].

**Table 1.** A summary of facilities available on the ISS to provide diverse biomedical experimentation

No	Facility	Scope	Supervision	Location	Reference
1	Advanced Biological Research System (ABRS)	Study of plants, microorganisms, and small arthropods (insects and spiders) growth	NASA	EXPRESS Rack 2	[62]
2	Aquatic Habitat (AQH)	Breeding small fresh-water medaka or zebrafish in special microaquaria	JAXA, Roscosmos	Multi purpose small payload rack (MSPR)	
3	Bioculture System Facility, Fig. 5b	Supporting short- and long-duration tests with living cells, microbes, and tissues with reconfigurable platform	NASA, Commercial	US Laboratory	
4	BioLab Experiment Facility, Fig. 5a	Experiments on microorganisms, cells, tissue cultures, small plants, and invertebrates	ESA	Columbus laboratory	
5	Biorisk	Study of bacteria/fungi adaptation limits and genotypical changes in space environments	Roscosmos	Pirs docking compartment	
6	Bone Densitometer, Fig. 5e	Measures of bone and muscle loss in rodents during spaceflight	NASA, Commercial	EXPRESS Rack 7	
7	Commercial Generic Bioprocessing Apparatus (CGBA)	Study of protein crystal growth, small insect habitats in a fully programmable and customizable incubator	NASA, Commercial	Columbus laboratory	
8	European Modular Cultivation System (EMCS)	Experiments on plant biology (cultivation, stimulation) in a reduced-gravity environment	ESA, NASA	EXPRESS Rack 3A	
9	Expose Experiment (Expose)	Short- and long-term experiments on exposure to space conditions and solar ultraviolet radiation in a field of exobiology and organic chemicals	ESA	Zvezda Service Module (Expose-R) and the European Columbus laboratory (Expose-E)	
10	JAXA Microscope Observation System (Fluorescence Microscope)	Biological observations on cell cultures and fish larva	JAXA	Multi Purpose small payload rack (MSPR)	
11	Kriogem-3 M, Fig. 5b	Refrigerator-incubator used for the storage of biological samples and medical kits	Roscosmos	Zvezda Service Module	
12	KUBIK, Fig. 5c	Thermal chamber functioning both as an incubator and a cooler	ESA	Zvezda Service Module	
13	Mouse Habitat Unit (MHU)	Investigation of rodent in integrated system with environment control and video recording system	JAXA	Cell Biology Experiment Facility (CBEF)	
14	Multipurpose Variable-g Platform (MVP)	Studies of model organisms and cell cultures with controlled lighting, gas/fluid exchange, and thermal and humidity control	NASA, Commercial	US Laboratory	
15	NanoRacks Astrium Centrifuge	Simulations of an artificial gravity environment (up to 1 g) focusing on biology and microbiology experiments	NASA, Commercial	US Laboratory	
16	NanoRacks Plate Reader, Fig. 5f	Real-time analysis in a field of biochemistry, molecular biology, cancer research, and immunology	NASA, Commercial	US Laboratory	
17	Osteoporosis Experiments on Orbit (Osteo-4), Fig. 5g	Study of bone cells growth in microgravity, in controlled environments	CSA	N/D	
18	Plant Habitat	Plant bioscience research in a fully automated, environmentally controlled chamber	NASA	JEM Kibo	
19	Rodent Research Hardware System, Fig. 5h	Containment system for rodents in support of biological studies	NASA	Microgravity Science Glovebox (MSG) facility	
20	Space Automated Bioproduct Laboratory (SABL)	Supporting experiments for development of applications for use on Earth by pharmaceutical, biotechnology and agribusiness companies	NASA	US Laboratory	
21	Space Technology and Advanced Research Systems – 1 Experiment Facility (STaARS-1 EF)	Facilitation of drug discovery, drug compound production and virulence novel modelling, supporting biomedical therapeutic markets	NASA, Commercial	US Laboratory	
22	Saibo Rack (Saibo)	Supporting cell cultures, plant cultures and mouse projects across a wide range of biological sciences	JAXA	JEM Kibo	
23	TangoLab-1, Fig. 5i	Experiments on human tissue regeneration, drug development and diseases treatments, e.g., cancer	NASA, Commercial	EXPRESS Rack 4	
24	TBU-N Low-temperature incubator	Refrigerator to conduct a variety of experiments in human life sciences, biology and biotechnology	Roscosmos	Mini-Research Module 1 (MRM1)	
25	TBU-V High-temperature incubator	Experiments in human life sciences, biology, and biotechnology at elevated temperatures	Roscosmos	Mini-Research Module 1 (MRM1)	
26	Vegetable Production System (Veggie)	Plant growth chamber supporting experiments on larger-sized plants	NASA	EXPRESS Rack 3	
27	WetLab-2	RT-PCR platform for gene expression analysis covering a variety of biospecimen types to be tested	NASA	US Laboratory

As shown in the Fig. 5, the facilities of ISS due to substantial room limitation are typically a cubic box type containers of small dimensions. For instance, KUBIK measures 37 cm^3^, similarly is in the case of Bioculture System Facility, which provides cartridges for 10 independently controlled bio research. Such small volume and the need for experiment automatization suggests the application of lab-on-chips. Thus, NASA and the National Center for Advancing Translational Sciences (NCATS) along with National Institute for Biomedical Imaging (NIBIB) and other scientific units have already started the project on development of microphysiological systems (MPS), so called “tissue chips” [63–66]. The main purpose of the program is to leverage unique microgravity environment of the ISS to model diseases on-chip and provide the knowledge on cells behaviour.

**Fig. 6:**
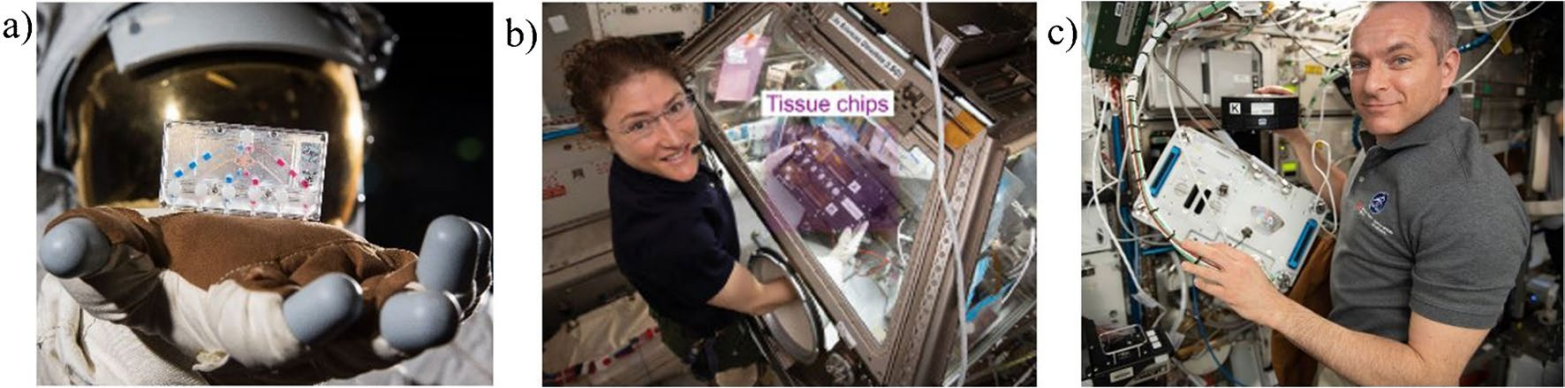
Tissue chips in ISS: **a** a NASA spacesuit with a kidney tissue chip in hand. Image courtesy of NASA, **b** Astronaut Christina Koch operating kidney chips [63], **c** Canadian astronaut David St-Jacques checking on the MIT cartilage-bone-synovium LOC platform. Image courtesy of NASA

To date, 9 projects have been funded by NCATS and NIBIB under the frames of “tissue chips,” coming into the scope of immune alterations, musculoskeletal deconditioning, kidney dysfunction, and cardiac tissues [63, 66–68], Fig. 6. The programs cover the development of LOC platforms and their adaptation for the spaceflight and approximate month-long experiment on the ISS.

One of the projects focuses on immune system response to microgravity in the context of immunosenescence. The team from University of California San Francisco (UCSF) works with T cells and more specifically, TEMRA cells, to investigate the potential cells vascular regeneration and bone healing. Other project connected with immune alterations is run by Children’s Hospital of Philadelphia. The researchers study lung bacterial infection (*Pseudomonas aeruginosa*) utilizing a lung-bone tissue chip. In a following project, the so called cartilage-bone-synovium LOC was proposed by the Massachusetts Institute of Technology to provide the knowledge on bone resorption and potential inflammatory responses to therapeutics. In turn, University of Florida develops a skeletal tissue chip that will be used for investigation of primary human myocytes from young, older, healthy and sedentary volunteers. The next project is related to kidney dysfunction in microgravity, namely the cell polarization of proximal and distal tubule epithelial cells will be investigated with simultaneous determination of vitamin D bioactivation and cells interaction. Stanford University proposed to develop, so called, engineered heart tissues (EHTs) for the spaceflight experiment. Specifically, human iPSC-derived cardiomyocytes will be investigated towards alteration in cardiac functioning. The tests assume also the drug-screening as a solution to prevent or reverse the microgravity induced cells changes. Similar experiments will be performed by University of Washington [66] that will use the same cell line in MPS platform characterized by extracellular scaffolding matrix with embedded electroconductive composites. The major goal of the research is to enhance the maturation process of cardiomyocytes. Two last project will be implemented by the Emulate company. One of the solution refers to the development of blood–brain barrier chip (BBB) to study neurons and vasculature expression during spaceflight-relevant stresses, e.g., hypoxia. The second proposal focuses on “barrier chip” investigating interactions and biological signals pathways between epithelial mucosa of the gut and neurons in conditions of *Salmonella typhimurium* infection.

All the aforementioned projects are ambitious and special attention has to be paid for the MPS durability and performance under harsh space environments. The solutions are still at their infancy and to the best knowledge of the author, none of the group has published to date the detailed experiment methodology, chip construction, technical and biomedical results. As described above, each of the project refers to radically different biomedical object, thus this initiative may accelerate the translation of the solutions and provide substantial improvements in Earth laboratory studies, too.

**Fig. 7:**
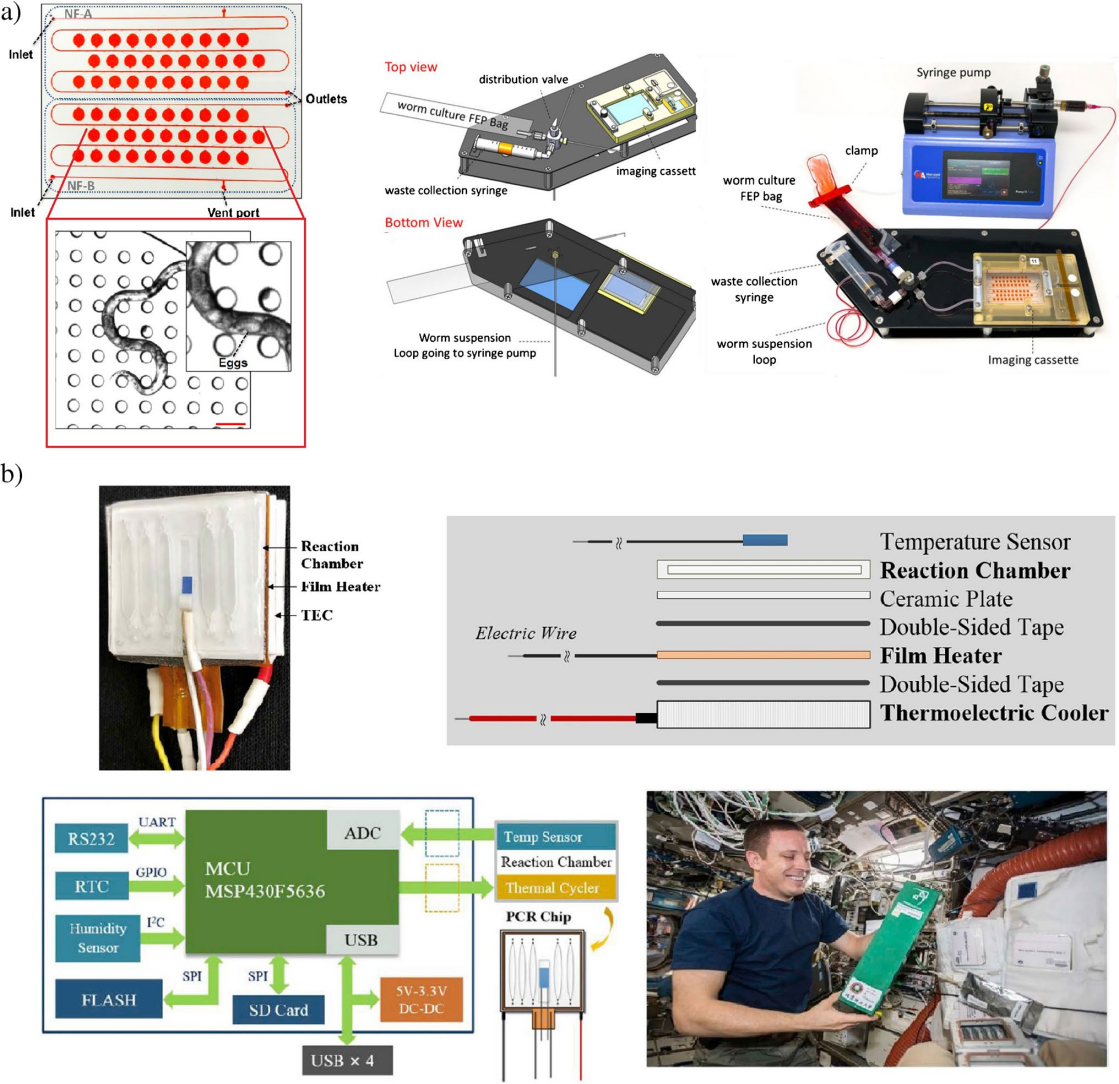
Examples of microfluidic platforms for biomedical research used in ISS: **a** NemaFlex device with magnified view of the C. elegans crawling the pillar, scale bar: 100 µm (on the left), scheme of the fully integrated microfluidic system (in the middle), real view of the NemaFlex microfluidic system (on the right) [69], **b** real view of the PCR chip (on the top left), exploded schematic view of the chip (on the right), schematic diagram of the control circuit (external power supply and data transfer of the device are provided through four USB ports. Each temperature sensor and thermal cycler of the twelve PCR chips are connected to the circuit board) (on the bottom left), NASA Astronaut Jack installing the device to the ISS platform [72]

Apart from the recent program of “tissue chips,” scientists around the world benefit with ESA and NASA programs for academia to implement the research in the ISS. In the paper [69], the so called, NemaFlex microfluidic device was proposed to measure behavioral changes of *C. elegans* in microgravity, especially with a view to muscle strength (Fig. 7a). The solution was an improvement over the previous NemaFlex system [70] (lab-chip was redesigned), and additional tools, i.e., flow management apparatus with control of microorganisms injection without channel clogging, bubble generation and fluid leakage, were proposed. As reported, substantial technical advances ensured to conduct spaceflight studies which showed that muscle-related genes face decreased expression in microgravity environment. The authors are still interested in further studies of this scope, namely to check, if the observed changes can persist across multiple generations or rather constitute a temporary mechanism.

Interesting solution was also reported in [71]. The so called Lab-On-a-Chip Application Development Portable Test System (LOCAD-PTS) was used to quantitatively indicate the presence of endotoxin in ISS cabins, which is a marker of Gram-negative bacteria and fungi. As a result, endotoxin was detected at 24 out of 42 surface areas at low, moderate, and high levels. High to moderate levels (1.01 to 14.7 endotoxin unit per 100 cm^2^) were found on 11 surface areas, e.g., at exercise, hygiene, sleeping, and dining facilities, which seems especially surprising in the context of the previous indications that no Gram-negative bacteria can be faced on ISS. The authors suggest some new regulations to provide both safeguarding crew health and monitoring forward contamination during space missions.

The use of microfluidic instruments is also more and more popular to perform genetic analyses abroad the ISS. C. Yang et al. fabricated a polymerase chain reactions (PCR) lab-on-chip system containing optical reaction chamber, miniaturized thermal cycler, and thermoelectric cooler (Fig. 7b) [72]. The system was based on a multi-channel approach — the device of five channels × twelve chips supported a number of 60 PCR performed simultaneously in a single chip utilizing gel electrophoresis method. 35 temperature cycles were run during a single PCR experiment. In each cycle three stages could be distinguished: denaturing (94 °C, 30 s), annealing (53 °C, 30 s), and extension (72 °C, 120 s). The experiments performed on ISS were related to investigation of cumulative radiation effect, thus two tests were conducted separately – one just after the start of on-orbit flight, the second 10 days after the first one. Thanks to temperature control on-chip, sensor data showed that all amplification experiments were completed as expected. The electropherogram analyzes of the both space and reference samples were performed on the ground and very initial results are shown in the paper. Electrophoresis results confirmed that all the genes used for the experiment were amplified, however further research needs to be done to appropriately perform all the sequencing and data analysis.

Apart from the aforementioned, self-developed analytical instruments, gene chips used in the ISS are very often commercially available solutions. One of the example can be MinION DNA sequencer developed by Oxford Nanopore Technologies (Oxford, UK) which was used for nanopore DNA sequencing and genome assembly of lambda bacteriophage, *Escherichia coli* and *Mus musculus* [73]. The tests were conducted over a 6-month period and no decrease in sequencing performance was observed over this time. The results of genome assemblies for each of the microorganisms confirmed feasibility of sequencing analysis and microbial identification aboard the ISS.

The aforementioned examples of LOC-based studies performed in the ISS however still constitute a minority in comparison to the traditional cell culture facilities. For instance, Kruger et al. established the cultures of endothelial cells (ECs, EA.hy926 cell line) in 75 cm^2^ tissue culture flasks to evaluate the microgravity influence under the ESA-SPHEROIDS campaign [74, 75]. Higashibata et al. reported on the use of polyethylene bag for *Caenorhabditis elegans* 4-day culture in the ISS [76]. Similarly, Fitzgerald et al. used Teflon culture bags for lymphoid tissue cultures [77]. In many of the cases, the analysis of the sample was done in the home laboratory, after the samples were returned from the ISS, which seems quite a disadvantage. Similar approach was presented in the paper [78], which reported on cyanobacterium (*Nostoc* sp. CCCryo 231–06) studies in the ISS for 23 months utilizing classical cultures methods. Next, microfluidic chip was used for the in-depth studies of the space radiation influence in the Earth laboratories by the single cell whole genome sequencing (SC-WGS) method.

Undoubtedly, the opportunity to conduct the experiment with simultaneous sample analysis in a real-time manner, directly in space environment, seems reasonable, not to lose any significant biological responses. As a consequence of return flight, some sample degradation can be faced, being a result of additional exposure to radiation, elevated temperatures, shocks/vibrations and time. This may provide undesired, combined investigation effect, not necessarily simply connected with microgravity and/or radiation influence. Lab-on-chip technology has a potential to meet all the requirements of the reliable space studies. The development of microfluidic systems for the purpose of biomedical and pharmacological research in microgravity is one of the most important part of NASA and ESA strategies [79, 80]. As mentioned, these experiments may have substantial implications for earthbound biomed- and biopharma- plans for development, too.

According to the papers [63, 68] in 2022–2025 timeline, circa eight culturing LOCs for investigation of diverse cells and tissue behavior in response to space environments will be developed and tested in the ISS (Table 2). Some of the experiments also assume simultaneous drug delivery research to promote the understanding of the molecular and cellular resistivity mechanisms towards evaluation of novel pharmaceutical targets [81].

**Table 2:**
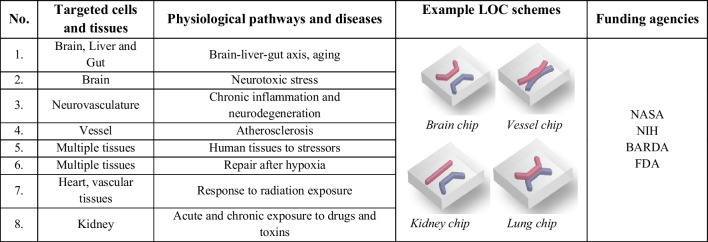
Cell and tissue chips under implementation for ISS microgravity research in 2022–2025, based on [63]

## LOC platforms as biological payload solutions for CubeSat satellites — missions accomplished

Prior to the description of the microfluidic platforms as the biological payloads for satellites missions, few examples of the miniaturized systems that found their application in different spacecrafts will be presented at first.

**Fig. 8:**
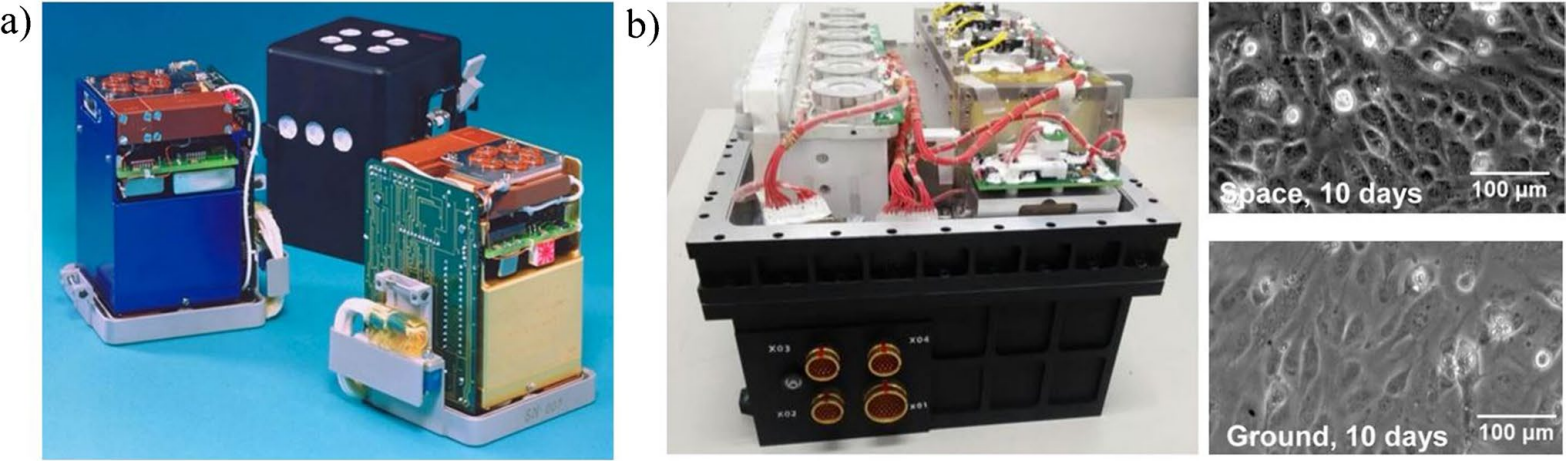
Images of **a** flight models of the Miniature Space Bioreactor. Image courtesy of ESA, **b** space cell culture system (SCCS) with example magnified view of EA.hy926 cells morphology cultured in space or on ground [84]

In the author’s opinion, pioneer research in this regard was, so called, Space Bioreactor [82]. Space Bioreactor was constructed under the frames of ESA PRODEX program in 1990 (Fig. 8a). Utilizing classical micromachining techniques of silicon and glass, the instrument ensuring cell cultivation research in space was fabricated. Space Bioreactor was designed to fit the dimensions of small container **—** 85 × 60 × 60 mm^3^. Apart from the silicon-glass microreactor, to control the supply of nutrition to the cell culturing chamber, the device also incorporated a media delivery system (silicon membrane pump and flow sensor). Moreover, pH, redox potential, and temperature sensors were applied to monitor the conditions prevailing in the instrument.

First experiments on Space Bioreactor were conducted in 1994 and 1996 as part of Space Shuttle missions **—** STS-65 and STS-76 [83]. The culture of yeasts was exposed to space environments, showing notable morphological changes during division process. The bioreactor was also planned to be used during Columbia mission in 2003 (STS-107); however, it was not accomplished.

In other paper [84], the space cell culture system (SCCS) was proposed as a payload of the SJ-10 satellite mission performed in 2016 (Fig. 8b). Although the solution did not use microfluidic chip directly, the SCCS was designed in-house to achieve a small scale laboratory device. Herein, two cell culture chambers providing 12 cm^2^ area for endothelial cells growth (EA.hy926) were assumed. MEMS pumps and pinch valves were used to refresh the cell cultures every 48 h and collect supernatant. Thanks to the use of silicon rubber seal in the culture chamber, the gas exchange preventing cells from hypoxia could be provided between the cell culturing medium and SCCS environment. The cells were maintained at the temperature of 36 °C. Automatic control of media delivery system also ensured to fix the samples with 4% paraformaldehyde at the end of the mission. Similar SCCS instrument was used for the reference ground experiments, conducted in strong agreement with the in-flight mission.

After 12 days of the experiment, the satellite was returned to the ground for in-depth studies. The effect of space microgravity on endothelial cells was analysed in the context of cellular morphology, metabolism, adhesive molecule expression, cytokine secretion, cytoskeletal remodeling, ECM accumulation, NO production, and the related mechanotransduction pathways. In general, space microgravity suppressed energy metabolism, cytokine secretion and ECM expression, modulated distinctly adhesive molecule expression, induced cytoskeletal remodeling, and enhanced exosome-mediated mRNA transfer. The scientists also found that not only microgravity may influence the cells growth, but also space radiation, hypergravity, and vibrations. This can result in a combined effect on cells, especially that some differences were observed in the outcomes achieved in simulated microgravity conditions, published elsewhere [85–87]. Authors underline that further research has to be done to clearly indicate the effects of space microgravity on ECs, as well as distinguish and understand the differences between real and simulated space influence.

Going back to the main path of the chapter, satellites of a CubeSat type have a growing potential to expand the microbiological research and provide more frequent space biology studies. Remotely controlled experiments utilizing CubeSats are of high concern now mainly due to scientific autonomy (no dependence on ISS astronaut crew), extended capability of research, and cut costs. CubeSat satellites are more and more often constructed in the micro (10–100 kg), nano (1–10 kg) and pico (0.1–1 kg) size. CubeSats are standardized with cubic modules expressed in units, where 1 U ≈ 10 × 10 × 10 cm, i.e., 1.33 kg. Microfluidic payload approach provides multiplication of experiments and thus reliability increase, whereas systems of MEMS/MOEMS sensors and actuators ensure automated and fully controlled experiment management. Below, all the biological CubeSat missions utilizing microfluidic systems that have been successfully launched are described and summarized in the Table 3.

**Table 3:**
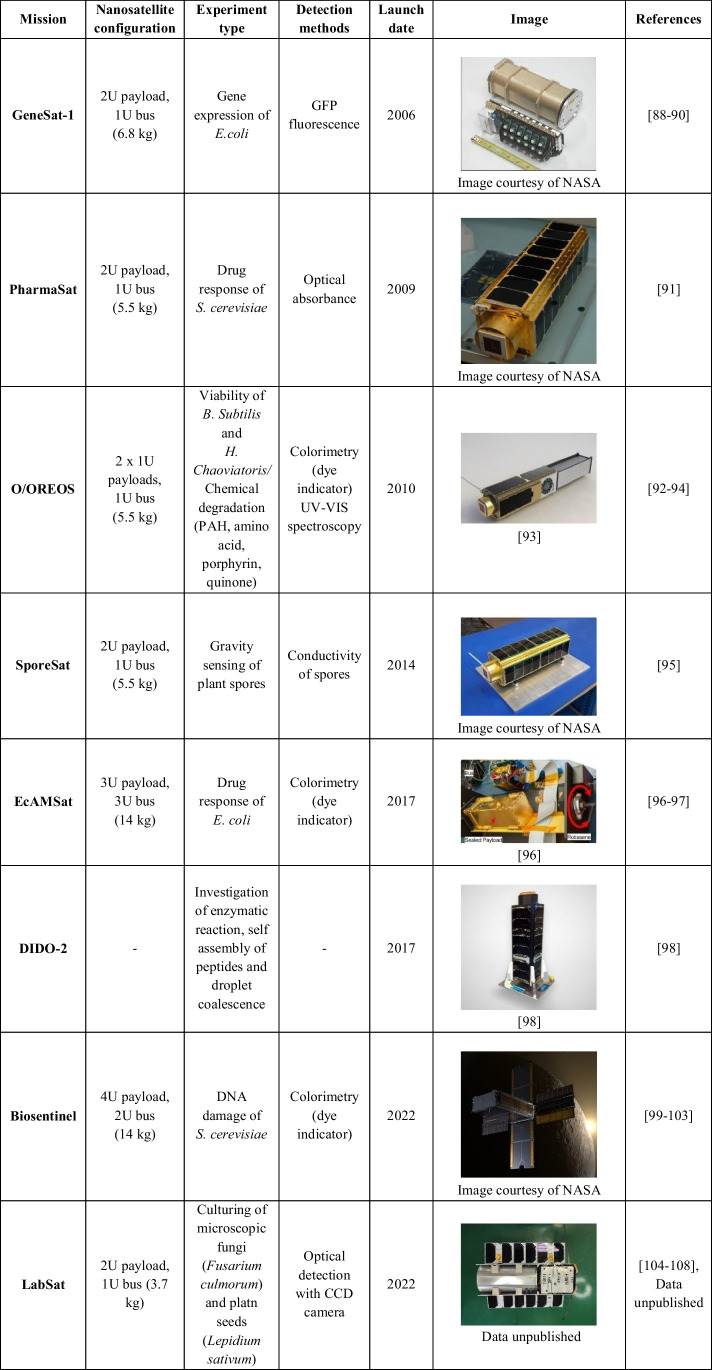
CubeSat type astrobiological missions — overview

**Fig. 9:**
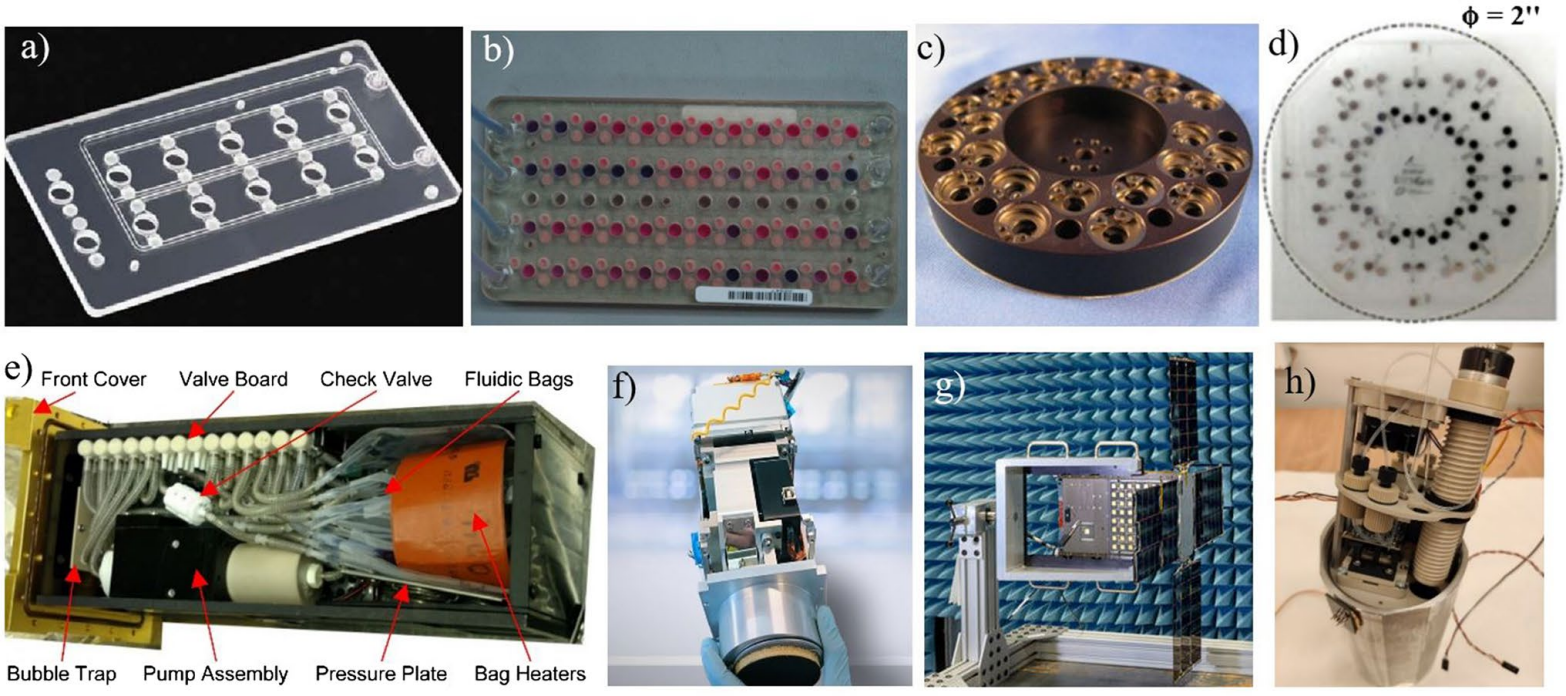
Images of microfluidic structures and integrated laboratory payloads of the bio-nanosatellites missions: **a** GeneSat-1 12-well microfluidic card. Image courtesy of NASA, **b** PharmaSat 48-well microfluidic card. Image courtesy of NASA, c) O/OREOS SEVO sample carrousel for handling 24 samples [93], **d** SporeSat bioCD with 32 sample wells [95], **e** EcamSat payload [96], **f** DIDO-2 payload [98],** g** Biosentinel flight unit during tests. Image courtesy of NASA, **h** LabSat payload (data unpublished)

The first mission of CubeSat with *life science* focus, GeneSat-1, was implemented in 2006 (Fig. 9a) [88, 89]. It was preliminary, real-time in situ measurement of microorganisms growth on Low-Earth-Orbit (LEO), with special attention paid to population growth and evaluation of *E. coli* bacteria gene expression. These well-known model organisms were cultured for 96 h in a payload containing 12-well microfluidic card. The viability of the cells was measured based on light scattering and fluorescence (GFP) method. With regard to diverse bacteria strains used for the test, different optical density and fluorescence signals were obtained. In most cases, a notable growth of *E. coli* specimens in microgravity was observed [90].

The following mission in LEO was realized in 2009 by PharmaSat (Fig. 9b) [91]. The main goal of the experiment was the investigation of microgravity effect on yeast susceptibility to antifungal drugs. Three different concentrations of drug were investigated to inhibit the growth of yeasts. The potential development of the fungi was indicated based on an optical absorbance with a focus put on a cell density as turbidity measurements. In this experiment, a 48-well card (similarly as in the previous mission) was used to provide cultivation chambers for the samples in a laboratory payload. Significant differences in metabolic activity of fungi in microgravity vs. terrestrial were observed, coming to the early findings that some pathogens can be more virulent in microgravity and can develop enhanced drug resistivity mechanisms.

Interesting LEO mission was also proposed by NASA in 2010 to study the biological response of bacteria and organic matter to orbital stresses — O/OREOS (Fig. 9c) [92–94]. That was a two-part experiment (SESLO and SEVO). In SESLO payload, the viability, growth rate and metabolism of *B. subtilis* of different origin (wild type and radiation-sensitive mutant) was investigated by in-situ colorimetry in a 12-well microfluidic card. Moreover, the influence of space radiation and microgravity on the selected biomarkers and organic molecules was evaluated utilizing ultraviolet and visible spectroscopy in SEVO payload. The preliminary results of the mission showed that the samples aboard O/OREOS germinated, grew, and metabolized significantly slower in comparison to ground-control samples.

The following LEO mission with *life science* focus was SporeSat (1 – 2014, 2 – 2017), which basic objective was to study mechanisms of plant cell gravity “sensing” (Fig. 9d) [95]. The model fern, *Ceratopteris richardii*, was used to determine the gravitational thresholds for calcium-ion channel activation of a single-cell spore. Herein, for the first time a lab-on-chip payload equipped with a special, fused-silica bioCD with a conductive layer was used for the measurement of the calcium-ion (Ca^2+^) channel activity at opposite ends of each spore. According to the mission results, the bioCD effectively recorded significant electrical signals in the spore, increasing with g-force, which were interpreted as gravity-directed calcium transport in the plants. In addition, some mutant plants were also developed during these studies, which for instance, were able to supress the expression of genes encoding Mechanosensitive Channel of Small Conductance (CrMscS).

Important results were also achieved in the EcAMSat LEO mission (2017) which aimed at the evaluation of antibiotic resistance of *E. coli* in microgravity conditions (Fig. 9e) [96]. The biological payload was prepared to maintain the bacteria culture at the elevated temperature and deliver necessary nutrition and antibiotic drugs. The payload was adapted from spare PharmaSat fluidic system, containing, e.g., microfluidic card, metering pump, bubble trap, and tubing connection. Cells growth and metabolic activity were evaluated based on optical detection system and LED illumination. Absorbance spectra at different wavelength (470 nm, 525 nm, and 615 nm) were measured to assess cells viability (based on metabolic indicator AlamarBlue). According to the papers [96, 97], the experiments showed that the studied cell strains were unaltered by microgravity. On the contrary, after treatment with gentamicin drug, the strains exhibited decreased metabolic activity, being consistent with some previous smallsat mission observations.

DIDO-2 was the mission proposed by SpacePharma company (Herzliya, Israel) (Fig. 9f). Although the satellite was launched in 2017, the information about the results is provided very sparingly, solely on the company website [98]. The research was focused on investigation of enzymatic reaction, self-assembly of peptides and droplet coalescence. Interesting outcomes of the studies showed that enzymatic reaction kinetics in microgravity was circa tenfolds slower in comparison to the earth conditions. In turn, phenylalanine peptides were found to change the structures towards spherulite-like in weightlessness state. When it comes to the droplet coalescence, a minimal drop coalescence occurred compared to immediate phase separation on earth. SpacePharma is also preparing DIDO-3 mission which will be aimed at investigation of microgravity effects on antimicrobial agents on bacterial pathogen and human serum albumin binding properties. However, no further information in this regard are provided.

Under the frames of Biosentinel mission (2022), to the best knowledge of the author, the first experimental approach to conduct biomedical experimentation beyond the Low-Earth-Orbit, in deep space environments, was proposed (Fig. 9g). The major focus herein was to monitor the DNA damage in response to radiation of modeler yeasts species, *S. cerevisiae*. Once the biological payload is in the orbit, the cells growth and metabolism are measured by optical detection system and metabolic dye indicator, similarly as in the previous solutions. The main motivation for this work is to evaluate the potential biological risks, as a measurable and parametrized factors influencing astronauts condition and health in deep space missions. The launch of the Biosentinel took place in November 2022. A duration of the experiment on radiation-induced DNA cells damage will cover about 6–12 months. As a control unit, the supporting biological payload will be additionally placed on ISS [99–103].

Last but not least is a LabSat mission, launched to LEO in 2022 (Fig. 9h) [104–107]. The microfluidic payload containing lab-on-chip instruments and life-support systems was developed to ensure long-term culturing of microscopic fungi (*Fusarium culmorum*) and cress seeds (*Lepidium sativum*) in microgravity conditions. The seed was cultured in a 3D printed micropot with capability to assess the quality of growth based on the mechanical root strength (measurement of the beam deflection) [107, 108]. Fungi in turn were investigated in all-glass LOCs, which construction is described in detail elsewhere [50]. Nutrition dosing units, as well as flex PCBs were applied to refresh the cultures and maintain appropriate culture temperature. White LED diode was used to illuminate the samples during the image acquisition. Optical detection system in a form of minicamera with CCD matrix and automatically adjusted focus ensured to capture the sequence of images. The microfluidic payload was integrated with CubeSat nanosatellite developed by SatRevolution company (Wroclaw, Poland). Unfortunately, after two weeks of the mission, the communication with CubeSat failed, thus few images of germinating cress seed could be solely taken.

Almost all of the aforementioned missions were developed by NASA Ames Research Center (ARC) [109, 110], except for DIDO-2 which was a commercial solution, and LabSat — fabricated by consortium partners — Wroclaw University of Science and Technology (Wroclaw, Poland) and SatRevolution company (Wroclaw, Poland). Miniaturization aspect is strongly visible in all the satellites covering structures of circa 3U-6U, small mass, limited low power and energy consumption. In each of the solution, a microfluidic chip was used for the biomedical experimentation. Typically, real-time optical detection methods for sample analysis were applied, i.e., fluorescence, colorimetry, spectroscopy, and CCD camera vision system. An iterative approach can also be noticed, especially with NASA solutions — subsequent missions took advantage from the previous ones, towards optimized payload structures. One of the most personalized LOC solution presented in a form different than microfluidic card can be considered bioCD proposed in SporeSat mission, and all-glass LOC and 3D printed micropot developed for LabSat mission. None of the CubeSat missions has been focused on human derived samples so far, i.e., stem cells, cancer cells. This is strictly related to prolonged satellite integration time, that has been mentioned in chapter 2 and article [51] of this paper. Fast payload integration (8 h prior to launch) was possible only in SCCS, mounted onto the SJ-10 platform. In other missions, few months (1–3 months) were needed which is a notable limitation for biomedical samples. Thus, this issue is of key importance to popularize the CubeSat space research on more sensitive bio-objects.

## Conclusions and future perspectives

Lab-on-chip microfluidic devices are maturing space applications, which may bring reliable and cost-effective science and exploration. Thanks to multi-functional and automatized LOC character, numerous opportunities for biomedical, biochemical and pharmaceutical studies can be raised. Novel microfluidic approaches, as well as adaptation of common microfluidic technologies is required to extend the LOC utility for space, especially that increasingly ambitious projects proposed by NASA and ESA may benefit from miniaturized and dependable instrumentation.

The major differences between Earth vs space LOC technologies are first and foremost related to the materials and fabrication procedures that can be applied in the aforementioned conditions. In order to extend the space microfluidic approach, LOC materials have to match both the low outgassing criterium and provide perfect structure encapsulation. It is not only connected to fluid leakage in zero gravity environment but also potential biological contamination, thus typically used polymers in terrestrial laboratories (mainly semi-permeable PDMS) will not be suitable in space. The solution to this problem can be the application of glass or thermoplastic materials accompanied by standard, well-known microengineering processes, i.e., chemical etching, hot embossing or bonding to achieve permanently joint LOC structures. Other focus should be put on mechanical durability of the microfluidic chips, as well as housing. Space LOC platforms must be resistant to vibration, acceleration and temperature changes. While for ground-based usage it is not critical, high precision components must be provided to protect the microdevices from damage and misalignment during space missions. Similarly, special shielding (e.g., aluminum cover) is needed to provide effective preservation of the samples from radiation. Space dedicated LOCs must be entirely autonomous, since little (ISS research) or no human control (satellite missions) is possible during experimentation. Although miniaturization aspect, low-weight and low-power are the features valid in all the LOC cases, for space instrumentation this becomes particularly important, determining costs and potential mission success. Possibly, ISS in the near future will organize special microfluidic laboratories to prepare LOC structures directly in space and on that basis, omit rough steps of the launch for the microscale instrumentation.

Microfluidics can play an important role for further space biology research, mainly in the context of CubeSats and ISS microgravity missions, deep space radiation analyses, but also ground-based microgravity simulations and short-term parabolic flights needing rapid detection of bio-sample response. Many microfluidic solutions have been recently studied on the ground, as well as utilizing microgravity simulators and special exposure chambers to assess the potential performance for future astrobiological missions. Some of the LOCs are ready to integrate with the satellite platforms, i.e., μFACS, μMAMA, worm imager. Miniaturized character of microfluidic instrumentation, low-weight and low-energy demands fits to the Starlight NASA roadmap program assuming to send small, fully autonomous spacecraft outside our solar system for in-depth behavioral analysis of well-known terrestrial microorganisms in microgravity and high radiation environments. Within this path, scientific institutions around the world propose new concepts of missions with life science focus, e.g., Minerva, GreenCube, SpectroCube, and AstroBio CubeSat.

Biomedical experimentation in space covers countless research areas; however, few majors directions can be distinguished. Main interest in this regard is put on the cells/tissues/microbials development in microgravity and/or radiation conditions (independently or in combination), biochemical reactions kinetics (e.g., protein crystal growth) and drug delivery research concerning both microfluidic phenomena and drug resistance of the bio-objects. In the ISS, the aforementioned biomedical research is conducted; however, there are still problems with broader accessibility, which is simply connected to limited ISS facilities, astronauts dependability, and thus, limited scientific opportunities. However, some ESA and NASA campaigns are provided to ensure scientists with the certain experiments. In this regard, “tissue chips” provided by NASA cover 9 programs that will investigate behavior of the certain cell lines with simultaneous diseases modeling. Application of microfluidic devices in ISS is more and more popular, however, these solutions are still a minority in comparison to standard laboratory techniques. In many cases, the biomedical samples are not analyzed in real-time manner, but studied thoroughly just after the return to the Earth. This approach may seem insufficient, especially with view to sample sensitivity to vibrations, changing temperature profiles, etc. Possibly, broader application of autonomous LOC platforms with integrated detection systems could solve the problem of delayed qualitative and quantitative sample analysis on the ground.

On the contrary to the ISS strategy, microfluidic systems were used for each of the accomplished astrobiological CubeSat experimentations. To date, 8 missions were launched, mainly operated by NASA AMES Research Centre, but this is the Miniature Space Bioreactor mission (1994, 1996: STS-65 and STS-76) run under the ESA PRODEX campaign that can be treated as the pioneer in the field of remotely controlled solutions utilizing microfluidic payloads. The missions with LOCs and MEMS components for biomedical research performed on CubeSat type satellites are: GeneSat-1 (2022), PharmaSat (2009), O/OREOS (2010), SporeSat (2014), EcAMSat (2017), DIDO-2 (2017), Biosentinel (2022), and LabSat (2022). Diverse microfluidic solutions (microfluidic cards, bioCD, 3D printed micropot, glass LOC, etc.) were employed with the detection methods limited to colorimetry, fluorescence, conductivity and minicamera vision system. Although many different bio-samples were investigated, e.g., bacteria (*E. coli, B. subtilis*), microscopic fungi, and plant seeds, none of the missions has been focused on human derived cells/tissues growth in space microgravity and radiation conditions so far. This is strictly related to the recent problems with time needed for the satellite integration and limited opportunities for the sample environment control prior to the mission launch. For this reason, the experiments are rather confined to less sensitive bio-objects, which can be prepared in dried form and do not need elevated temperature and media refreshment as the life-support. This problem forms a niche for specialized microsystem tools that could be used as intelligent zero-energetic systems ensuring appropriate incubation of the cells during waiting for the satellite launch. Other path may be connected with high-performance cryogenic platforms allowing for reliable maintenance of freeze-dried samples on-chip. These issues, however, require new miniaturized systems and bio-methodologies working in balance, since to the best knowledge of the author, on-chip human cells vitrification and thawing have not been performed to date.

As the developing of LOC technologies for space biomedical research, fulfilling all the requirements, is not trivial, close cooperation between scientists specializing in MEMS, microfluidic, and biomedical sciences, as well as space agencies members is essential. This may allow for acceleration in the field of both extreme space science and ground-based laboratory research. The translation of space biology towards development of new Earth therapies seems extremely realistic vision. Especially the aspects of microgravity-based cancer treatment have put a new light and a new hope onto multidrug resistant cell lines, mainly breast and thyroid cancer.

Although the bio-microfluidic platforms for ISS and CubeSat experimentation seem the major path within space biology field, biomarkers identification utilizing MEMS-based spectrometer instrumentation, mainly for the purpose of deep space missions, is also under the interest of international space agencies. MEMS components and microengineering techniques can also play an important role in development of ground-based microgravity simulators and multi-parametric exposure and sensory chambers, imitating interstellar environments. All the aforementioned issues chart possible future directions of the research.
