# Correction to: Lab-on-chip technologies for space research — current trends and prospects

**DOI:** 10.1007/s00604-023-06170-7

**Published:** 2024-01-10

**Authors:** Agnieszka Krakos

**Affiliations:** Department of Microsystems, Wroclaw University of Science and Technology, Janiszewskiego 11/17, 50-372 Wroclaw, Poland

**Correction to: Microchimica Acta (2023) 191:31**

10.1007/s00604-023-06084-4

In this article the author’s name Agnieszka Krakos was incorrectly written as Agnieszka Krakos (Podwin).

The original article has been corrected.

